# Supplementary material for: End Sequence Analysis Toolkit (ESAT) expands the extractable information from single-cell RNA-seq data
Source: Genome Res. 2016 Oct;26(10):1397–410. doi: 10.1101/gr.207902.116 (PMC5052061; doi:10.1101/gr.207902.116)
Supplement: Supplemental Material [file supp_gr.207902.116_Supp_Materials.tar.gz › ESAT-esat_v0.1/doc/html/classjaligner_1_1formats_1_1_pair.html]

ESAT: jaligner.formats.Pair Class Reference


ESAT


- Main Page
- Related Pages
- Packages
- Classes
- Files

- Class List
- Class Index
- Class Hierarchy
- Class Members

All Classes Namespaces Files Functions Variables Enumerator Pages

- jaligner
- formats
- Pair

Public Member Functions |
List of all members

jaligner.formats.Pair Class Reference

Inheritance diagram for jaligner.formats.Pair:

[legend]

Collaboration diagram for jaligner.formats.Pair:

[legend]

|  |  |
| --- | --- |
| Public Member Functions | |
|  | Pair () |
|  | |
| String | format (Alignment alignment) |
|  | |
| Public Member Functions inherited from jaligner.formats.Format | |
| abstract String | format (Alignment alignment) |
|  | |
| void | setId (String id) |
|  | |
| String | getId () |
|  | |

## Detailed Description

Pair format.

Author
:   Ahmed Moustafa (ahmed.nosp@m.@use.nosp@m.rs.sf.nosp@m..net)

## Constructor & Destructor Documentation

|  |  |  |  |  |
| --- | --- | --- | --- | --- |
| jaligner.formats.Pair.Pair | ( |  | ) |  |

Constructor

Here is the call graph for this function:

## Member Function Documentation

|  |  |  |  |  |  |
| --- | --- | --- | --- | --- | --- |
| String jaligner.formats.Pair.format | ( | Alignment | *alignment* | ) |  |

Formats an alignment object to the Pair\_FORMAT format

Parameters
:   |  |  |
    | --- | --- |
    | alignment | alignment object to be formated |

Returns
:   string of the alignment pair-formatted

Here is the call graph for this function:

---

The documentation for this class was generated from the following file:

- java/jaligner/formats/Pair.java


---

Generated on Fri Aug 15 2014 15:11:06 for ESAT by  

 1.8.7
